# Civil medical liability in general surgery: A systematic review

**DOI:** 10.1016/j.sopen.2026.05.001

**Published:** 2026-05-13

**Authors:** Ioannis Ketsekioulafis, Konstantinos Katsos, Christoforos Kolentinis, Theofano Sapka, Chara Spiliopoulou, Theodoros Lytras, Emmanouil I. Sakelliadis, Nikolaos Arkadopoulos

**Affiliations:** a Department of Forensic Medicine and Toxicology, School of Medicine, National and Kapodistrian University of Athens, Athens, Greece; b Air Force Hospital of Athens, Forensic Service, Athens, Greece; c Faculty of Law, National and Kapodistrian University of Athens, Athens, Greece; d 4th Surgical Department, Attikon University Hospital, School of Medicine, National and Kapodistrian University of Athens, Athens, Greece

**Keywords:** Medical liability, General surgery, Surgical malpractice, Systematic review, Malpractice claims

## Abstract

Civil medical liability is an important issue in modern surgical practice, affecting both medical practice and the doctor-patient relationship, and contributing to the increasing use of defensive medicine. General surgery, as a predominantly invasive specialty with a wide range of emergency and elective procedures, is at increased risk of legal claims. The purpose of this systematic review was to investigate the international literature on the civil medical liability of general surgeons, with an emphasis on the types of procedures involved, the causes of lawsuits, and the reported compensation amounts.

A systematic search of PubMed and Scopus was performed according to PRISMA guidelines, without geographical restrictions, and included only English-language studies. After removing duplicates and evaluating the titles, abstracts, and full texts, 41 studies were included in the final analysis.

Legal claims in general surgery typically involve common procedures such as cholecystectomy, hernia repair, thyroidectomy, and bariatric surgery. Compensation varies widely by country, with the highest amounts linked to permanent injury or death. The main causes include surgical errors, delayed diagnosis or treatment, missed postoperative complications, and poor patient consent documentation.

The findings suggest that medical liability in general surgery is a multifactorial issue, in which the management of complications and patient communication play roles as important as surgical technique. A systematic understanding of the factors leading to legal claims may contribute to improving patient safety, reducing defensive medicine, and enhancing the quality of surgical care.

## Highlights

• Medical tort liability is a significant issue in general surgery.
• Most claims involve common procedures, due to delays, errors, and poor care.
• Knowing claim patterns helps improve safety, care quality, and limit defensive care.
• European studies showed compensation maxima ranging up to €1,724,000.
• US data showed median payouts ∼$984,500 and verdicts ∼$2,000,000 with a wide IQR.
• UK settlements ranged widely, from about £5,000 to £470,000.

## Introduction

Medical liability is a fundamental pillar of medical law, as it sets the framework for the provision of medical services and regulates the rights and obligations of both health professionals and patients. Deviation from generally accepted standards of medical practice, when it results in patient harm, may give rise to civil claims for compensation, with significant professional, social, and economic consequences. The fear of possible legal involvement has been associated with the development of the so-called “defensive medicine”, in which clinical decisions are influenced not only by medical indications but also by the need to avoid legal consequences. This practice affects the quality of care provided, the doctor-patient relationship, and significantly burdens health systems [1], [2], [3], [4].

In recent years, the increasing frequency of medical lawsuits and the broader implications of medical liability have led to more intensive study of trends across different medical specialties. Comparative studies have shown that certain branches of medicine are at increased risk of legal disputes, particularly those involving invasive procedures and critical decision-making under time pressure. General surgery is a typical example of such a specialty, as it encompasses a wide range of interventions, from emergency to elective procedures, with the complication risk varying substantially according to the type, complexity, and urgency of the procedure, even under optimal medical conditions.

Civil liability claims against general surgeons often involve delayed diagnosis or treatment, technical errors during surgery, injuries to adjacent anatomical structures, postoperative complications, and insufficient information or documentation of the patient's consent [5], [6], [7], [8]. At the same time, the heterogeneity of surgical subspecialties and applied techniques leads to significant variation in both the causes of legal action and the amounts of compensation awarded. However, the available data remain fragmented, primarily drawn from individual retrospective studies or national legal systems, lacking a comprehensive synthesis of the findings.

To the authors' knowledge, there is no systematic review to date that collects and comprehensively analyzes the international literature on the evolution of civil medical liability in general surgery, the most frequent causes of lawsuits, and the reported amounts of compensation.

The purpose of this systematic review is to investigate the available literature from recent years to capture the impact of civil medical liability on general surgeons, identify the main causes of legal claims, and assess the overall financial compensation awarded. Understanding these parameters is expected to contribute to a more complete understanding of the phenomenon, enhance patient safety, and reduce the need for defensive medicine practices by improving clinical and organizational practices.

## Materials and methods

This systematic review was conducted in accordance with the PRISMA (Preferred Reporting Items for Systematic Reviews and Meta-Analyses) guidelines, with the aim of ensuring completeness and transparency in the process of searching and selecting the relevant literature (9). The search for published studies was conducted using PubMed and Scopus. There were no regional or chronological constraints in our search, and all included papers were written in English.

The search strategy combined terms related to the general surgical specialty with concepts related to medical liability, medical negligence, and legal claims. The terms used in the search were the following: (“general surgery” OR “general surgeon” OR “general surgeons” OR surgeon OR surgeons OR “abdominal surgery” OR “gastrointestinal surgery” OR “digestive surgery” OR appendectomy OR appendicectomy OR cholecystectomy OR colectomy OR “colorectal surgery” OR “hernia repair” OR hernioplasty OR laparotomy OR laparoscopy OR “emergency surgery”) AND (litigation OR malpractice OR negligence OR “medical liability” OR “professional liability” OR “medical error” OR “legal claim” OR “legal claims” OR lawsuit OR lawsuits OR “medical negligence”).

The bibliographic references of the selected articles were also examined to identify relevant publications not identified during the initial search. At the same time, a PubMed search was conducted for studies that cited the already selected articles, and the corresponding abstracts and full texts were evaluated as deemed necessary. The literature search was conducted during the initial study design and was updated until the final data collection phase.

The aim of the study was to compile all available scientific data on civil medical liability among general surgeons in recent years, analyze the factors leading to lawsuits, and evaluate their outcomes, including the compensation awarded. Studies that did not concern the general surgical specialty, were not related to medical liability issues, had been published in a language other than English, or did not provide information on the causes, incidence, or financial consequences of legal claims, were excluded from the review.

After removing duplicate publications, all articles were initially screened based on their titles and abstracts. Those deemed potentially suitable were then evaluated by reading the full text. The selection process was conducted independently by multiple researchers, and any disagreements were resolved through discussion and consensus. The authors used Rayaan for this systematic review (10).

Data extracted from each study included the names of the authors, year of publication, country or geographical area of study, type of surgical procedure, the causes leading to medical liability of general surgeons, and reported amounts of compensation where available. All information was recorded in a standardized electronic database.

Due to the qualitative and descriptive nature of the included studies, as well as the significant heterogeneity in study design, legal systems, and reporting of results, no meta-analysis or statistical synthesis was possible. Therefore, a descriptive analysis of the data was performed to identify recurring patterns and trends in civil medical liability in general surgery.

This systematic review was not registered in PROSPERO as the review question did not involve a healthcare intervention, which is currently required for PROSPERO registration.

Artificial intelligence–based language assistance tools were used only after the authors had completed the literature search, study selection, data extraction, synthesis of findings, and interpretation. Their use was limited to linguistic editing, improvement of grammar and readability, and refinement of phrasing in the manuscript draft. Specifically, ChatGPT (OpenAI) (11) and Grammarly (12) were used solely for text refinement after the authors completed the scientific content. No AI tool was used to identify studies, extract data, evaluate eligibility, appraise article quality, perform analysis, or generate scientific conclusions. All intellectual content, data interpretation, and final manuscript approval remain the sole responsibility of the authors.

## Results

### Literature review

A systematic search of the international literature was conducted in PubMed and Scopus to identify studies on civil medical liability in general surgery. In total, 14,353 articles were identified, of which 8212 came from PubMed and 6141 from Scopus. After removing duplicate publications, 10,940 articles remained, which were evaluated based on their titles and abstracts.

After the initial screening, 79 studies were deemed potentially eligible and were reviewed in full text. Articles that did not meet the inclusion criteria were excluded due to non-relevance to the general surgical specialty, absence of data on medical liability issues, or lack of information regarding the causes and outcomes of legal claims.

Following full-text review, a total of 41 studies were deemed relevant to the subject and were included in this systematic review. Finally, quality control was performed using the Joanna Briggs Institute (JBI) critical appraisal tools (13). Using these tools, no articles were excluded.

**Fig. 1:**
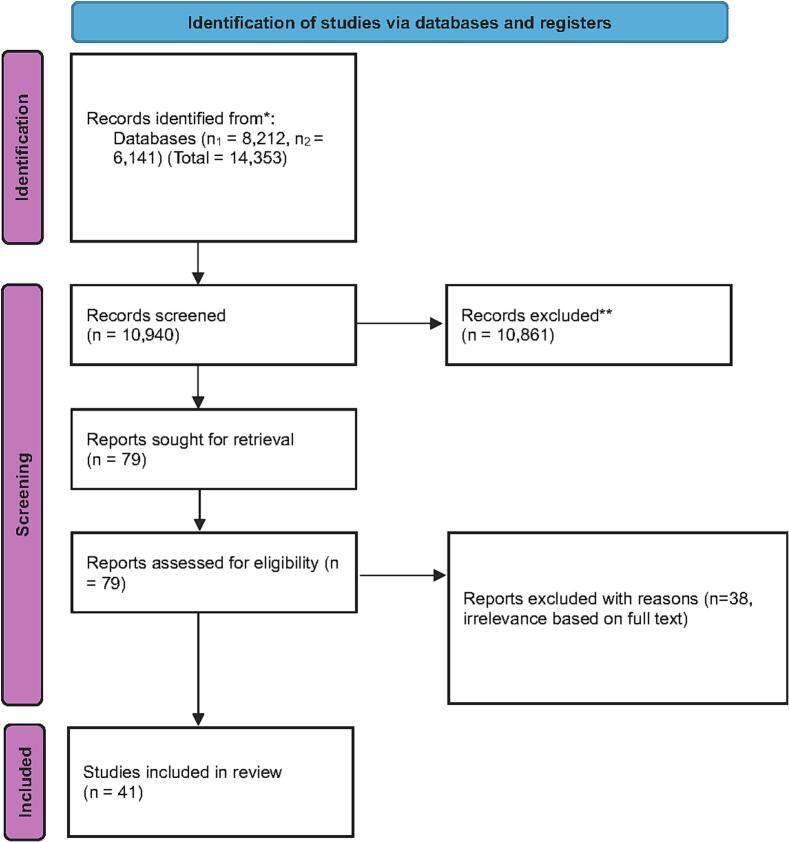
PRISMA flow diagram. n_1_ = Records identified from PubMed Database. n_2_ = Records identified from Scopus Database.

The study selection process is illustrated in detail in the PRISMA flow chart (Fig. 1).

The included studies were from different geographical regions worldwide, highlighting the international nature of civil medical liability in general surgery. The key characteristics and main findings of the selected studies are summarized in Table 1.

**Table 1:** Papers included in the systematic review.

Authors	Year	Region	Type of surgery	Compensation payouts	Causes
Cottam et al. (14)	2007	USA	Bariatric surgery	NS	Intestinal leak; Intra-abdominal abscess; Bowel obstruction; Major airway complications; Organ injury; Pulmonary embolism; Delay in diagnosis; Misinterpretation of vital signs (failure to recognize tachycardia); Misinterpretation of imaging studies (UGI contrast study, CT abdomen, chest X-ray); Inadequate prophylaxis for thromboembolism; Technical surgical error; Failure of care transition (“dropped baton” phenomenon)
Gambardella et al. (15)	2017	Italy	Thyroid surgery	Median jury award (USA literature review): $974,625; Institutional data (Italy): ∼€50,000 compensation for permanent unilateral vocal fold palsy; ∼€200,000 compensation for permanent bilateral vocal fold palsy	Recurrent laryngeal nerve injury; Vocal cord paralysis; Neural traction injury; Thermal injury; Clamping injury; Ligature entrapment; Ischemic nerve injury; Failure of informed consent; Inadequate surgical documentation; Delayed communication of complication
Ghislain et al. (16)	2019	France	Colorectal surgery	NS	Anastomotic leak; Injury to neighboring organs (ureteral, intestinal, vascular injuries); Bowel obstruction; Hemorrhage; Retained foreign body; Surgical technical error; Therapeutic delay; Delay in diagnosis; Delay in re-operation; Delay in transfer; Delay in imaging or laboratory evaluation; Inadequate postoperative monitoring; Failure of team coordination; Inadequate antibiotic therapy; Erroneous surgical indication; Inadequate diagnostic assessment; Poor medical documentation; Defective informed consent; Failure of cancer follow-up; Stoma management errors
Cardin et al. (17)	2019	France	Hernia repair	Transactional settlements up to €275,000; Highest liability remuneration reported: €645,000; NHS litigation data (comparative): Testicular injury ∼€50,084 average payout; Chronic pain ∼€87,674; Visceral injury ∼€69,526; Wound infection ∼€40,624; Sexual dysfunction ∼€92,177; Vascular injury ∼€36,134; Recurrence ∼€47,020; Hematoma ∼€24,387; Retained foreign body ∼€28,397; Wrong-site surgery ∼€38,436	Testicular injury; Chronic pain; Visceral injury; Wound infection; Sexual dysfunction; Vascular injury; Recurrence; Hematoma; Retained foreign body; Wrong-site surgery
Timsit et al. (18)	2019	France	Bariatric surgery	Highest compensation payout reported: €1,724,000 (ongoing indemnification case); Multiple insurer compensations and ONIAM liability decisions reported (amounts not systematically disclosed)	Anastomotic/staple-line leak; Visceral perforation (stomach, bowel, pancreas, colon, ureter); Hemorrhage; Infection; Bowel obstruction; Retained foreign body; Pulmonary embolism; Metabolic deficiency (Wernicke encephalopathy); Delay in management; Delay in imaging; Delay in transfer; Surgical technical error; Incorrect surgical indication; Inadequate patient information; Poor preoperative assessment; Inadequate follow-up after discharge; Failure to review test results; Nosocomial infection; Lack of multidisciplinary coordination
Delaunay et al. (19)	2019	France	General surgery (bariatric; colorectal; parietal; cholecystectomy; appendectomy; small intestinal; upper GI surgery)	Compensation €81,000 (bile duct injury); €30,000 (fatal hemoperitoneum after appendectomy); Maximum High Court compensation €1,421,636; Administrative court compensation €240,953.68	Nosocomial infection; Anastomotic leak; Poor postoperative management; Hollow organ perforation; Retained foreign body; Delay in reoperation; Poor operative technique; Incomplete operation; Bile duct injury; Hemoperitoneum; Peripheral neurologic injury; Insufficient preoperative information; Incorrect indication; Diagnostic error
Facchin et al. (20)	2023	Italy	Post-bariatric body contouring surgery (abdominoplasty; brachioplasty; reduction mammoplasty; liposuction; torsoplasty)	NS	Major complications (seroma relapse after abdominoplasty; brachial plexus injury after brachioplasty; skin necrosis after reduction mammoplasty); Minor complications (poor cosmetic outcome; hypertrophic scarring; scar malposition); Misinterpretation of informed consent; Unrealistic patient expectations
Eş et al. (21)	2017	Turkey	General surgery (gallbladder; thyroid; appendectomy; hernia; colorectal; breast; gastric-esophageal; intestinal; hepatic; trauma surgery)	NS	Lack of attention/care/skill during operation; Diagnostic error; Failure to recognize postoperative complication; Delay in treatment; Wrong operative technique; Indication error; Retained foreign body; Wrong-site surgery; Inadequate preoperative testing; Inadequate postoperative follow-up
Nurminen et al. (22)	2024	Finland	Antireflux surgery; Paraesophageal hernia repair	NS	Esophageal perforation; Gastric perforation; Intestinal perforation; Dysphagia; Bleeding; Vagal nerve injury; *Re*-herniation; Treatment failure; Need for reoperation; Severe postoperative complication; Long-term disability; Inadequate preoperative evaluation; Inappropriate surgical indication
Desserud et al. (23)	2017	Norway	Gastroenteropancreatic neuroendocrine tumor management (GI surgical oncology)	Compensation awarded in 4/7 cases; Range NOK 50,000–1,000,000; Median NOK 500,000 (∼€52,400)	Delayed diagnosis; Misinterpretation of symptoms; Failure to follow treatment guidelines; Inappropriate medical therapy; Diagnostic delay leading to disease progression
Varley et al. (24)	2020	UK	Hernia repair	Total payouts: £33.2 million overall; Laparoscopic £4.1 M (mean £82,824); Open £9.4 M (mean £66,796); Mean successful claim £78,715; Highest mean payouts associated with visceral injury (£116,482 lap; £199,103 open) and vascular injury	Operative error; Visceral injury; Vascular injury; Nerve injury; Testicular complications; Chronic pain; Wound infection; Hematoma; Retained foreign body; Wrong-site surgery; Delay in recognition of complication; Consent issues; Substandard postoperative care
Padovano et al. (25)	2022	Italy	Thyroid surgery	€261,883 total payouts; Mean €32,735 per compensated claim (8 settled cases)	Incorrect treatment; Recurrent laryngeal nerve injury; Incomplete thyroid removal; Permanent hypoparathyroidism; Aesthetic damage; Dental injury during intubation; Inadequate informed consent; Poor surgical report documentation
Moreira et al. (26)	2013	Portugal	General surgery (HPB; colorectal; gastric; abdominal wall; emergency surgery)	NS	Bile duct injury; Bowel injury; Major vascular injury; Diagnostic failure; Failure to perform indicated surgery; Delay in diagnosis; Postoperative sepsis; Hypovolemic shock; Inadequate clinical assessment; Possible violation of leges artis
Lindmark et al. (27)	2018	Sweden	Hernia repair	NS	Inadvertent enterotomy; Surgical site infection; Poor cosmetic outcome; Recurrence; Chronic pain; Mesh complication; Seroma; Ileus; Bleeding; Fistula; Organ injury; Negative exploration; Missed hernia; Anesthetic injury (dental injury; nerve injury; positioning injury); Inadequate preoperative assessment
Kern KA (28)	1997	USA	Cholecystectomy	Mean settlement payment: $469,711; Mean plaintiff jury verdict payment: $188,772; Mean payout all injuries combined: $437,822 ± $320,000 (range $95,000–$1,097,000); Total liability payout: $9,679,586; Mean bile duct injury settlement: $506,538 ± $307,000 (range $175,000–$1,000,000); Plaintiff verdict bile duct injury: $250,000; Mean bowel injury settlement: $437,500 (range $225,000–$650,000); Plaintiff verdict bowel injury: $248,547; Mean payout deaths from bowel injury: $536,750 ± $430,000 (range $175,000–$1,097,000); Total bile duct injury payouts: $6,585,000 (mean $488,214); Total bowel injury payouts: $1,123,547 (mean $374,516); Total vascular injury payouts: $256,539 (mean $128,270); Other injury payouts: $1,417,000 (mean $472,333)	Common bile duct transection; Common bile duct excision; Common hepatic duct transection; Clip ligation injury; Hilar hepatic duct injury; Cystic duct leak; Small bowel trocar perforation; Small bowel cautery injury; Duodenal cautery injury; Colon cautery injury; Unrecognized bowel injury with septic peritonitis; Iliac artery trocar injury; Iliac vein injury; Aortic trocar puncture; Bile spill with peritonitis; Retained intra-abdominal gallstones; Cautery burn injury; Postoperative hemorrhage with cerebral anoxia; Delayed hemobilia after conversion to open surgery
Schofield PF (29)	1999	UK	Colorectal surgery; Anal surgery;	NS	Faecal incontinence after childbirth injury; Faecal incontinence after haemorrhoidectomy; Faecal incontinence after fistula surgery; Faecal incontinence after anal dilatation; Anal pain after surgery; Postoperative anal infection; Complications after haemorrhoid injection; Symptoms after sigmoidoscopy; Diagnostic error; Error of consent; Failure to warn about impotence in pelvic surgery; Incorrect action after biopsy report; Delay in treatment of diverticulitis; Delay in treatment of appendicitis; Delay in treatment of inflammatory bowel disease; Delay in surgery leading to fatal outcome; Infertility allegation after appendicitis delay; Intestinal perforation during laparoscopy; Bleeding during laparoscopy; Major vascular injury during laparoscopy; Operative error; Colonoscopic perforation; Colonoscopic bleeding; Diagnostic failure at colonoscopy; Ureteric injury; Retained swab; Compartment syndrome after pelvic surgery; Bed sores; Postoperative bleeding; Colostomy closure complication; Faecal fistula; Wound infection; Wound hematoma; Wound pain; Incisional hernia; Excessive scarring; Wound sinus; Small bowel obstruction after surgery; Radiation bowel disease mismanagement; Bacterial endocarditis after operation; Bladder stone caused by pelvic suture
Panuganti PL et al. (30)	2020	USA	Colorectal surgery	Plaintiff payment range (failure to perform colonoscopy per screening guidelines): $135,000–$7,100,000; Mean payment $2,063,547.33 ± $2,601,614.30; Plaintiff payment range (failure to perform colonoscopy according to presenting symptoms): $0–$5,363,499; Mean payment $1,939,454.45 ± $1,901,506.44; Plaintiff payment range (failure to detect CRC with colonoscopy): $355,500–$3,975,000; Mean payment $1,683,055.80 ± $1,510,788.94; Plaintiff-verdict cases with metastasis: $0–$6,000,000 (mean $2,403,341.82 ± $1,868,863.25); Plaintiff-verdict cases with new cancer diagnosis: $135,000–$15,000,000 (mean $2,069,353.56 ± $4,142,430.24); Plaintiff-verdict death cases: $0–$7,100,000 (mean $1,791,894.40 ± $2,183,665.87); Plaintiff-verdict non-death cases: $150,000–$15,000,000 (mean $2,449,404.20 ± $3,636,742.78)	Failure to perform colonoscopy according to symptoms; Failure to perform screening colonoscopy according to guidelines; Failure to detect colorectal cancer during colonoscopy; Failure to detect colorectal cancer using non-colonoscopy diagnostic methods (CT; barium enema); Diagnostic error; Delay in diagnosis; Injury from surgery; Negligent surgery; Colonoscopy injury; Negligent colonoscopy; Retained foreign body during surgery; Failure of follow-up; Inadequate post-diagnostic care; Communication failure between providers
Dent PC; Bagnall NM (31)	2017	UK	Thyroid surgery	Total successful claims payout: £7,649,963.69; Average payout per successful closed claim: £99,350.18; Diagnosis delay: mean £61,106.14 (range £4496.04–£550,745.00); Incorrect diagnosis: mean £121,886.10 (range £9325.00–£1,450,005.26); Failure of consent: £134,046.73; Consent taken by non-operating surgeon: £73,661.85; Awareness during anaesthesia: £623,161.80; Diathermy burns: mean £9265.74 (range £7354.23–£11,243.00); Recurrent laryngeal nerve injury: mean £137,717.26 (range £8317.50–£945,685.26); Other nerve injury: £177,177.90; Low serum calcium: mean £23,294.69 (range £4512.50–£42,076.88); Swelling: mean £63,802.17 (range £11,534.29–£116,070.05); Poor postoperative care: mean £138,598.07 (range £26,888.22–£435,843.56); Scarring: mean £15,636.97 (range £8156.44–£23,117.50); Negligent treatment: £66,918.11; Never events: mean £22,672.76 (range £3393.59–£77,960.50); Loss of specimen: £3393.59; Thyroid gland removed in error: £7500.00; Swab left in wound: £8146.25; Incorrect surgery: £16,647.31; Inappropriate surgery: £22,388.90; Death following biopsy: £77,960.50	Diagnosis delay; Incorrect diagnosis; Failure of informed consent; Consent obtained by different clinician; Awareness during anaesthesia; Diathermy burn injury; Recurrent laryngeal nerve injury; Other nerve injury; Removal of incorrect tissue; Other surgical problems; Postoperative hypocalcaemia; Wound infection; Postoperative swelling/hematoma; Poor postoperative care; Scarring; Negligent treatment; Retained foreign body; Death following tracheostomy; Loss of histological specimen; Thyroid gland removed in error; Swab left in wound; Incorrect surgery; Inappropriate surgery; Death following biopsy
Jiam NTL et al. (32)	2014	USA	General surgery (procedure-related surgical malpractice across specialties reported in NPDB)	Total inflation-adjusted payout (1990–2011): $5,866,435,277; Average payout per claim: $301,259; Annual payout average: $266,656,149; Total payouts permanent injury: $4,717,009,542; Total payouts temporary injury: $1,118,765,668; Total payouts emotional injury: $30,660,067; Average payout permanent injury (delay in performance): $427,428; Temporary injury: $198,578; Emotional injury: $18,603; Improper performance average payout: $411,829 (permanent), $207,613 (temporary), $63,395 (emotional); Failure to recognize complication average payout: $409,713 (permanent), $215,232 (temporary), $40,955 (emotional); Improper technique average payout: $397,500 (permanent), $159,855 (temporary), $53,623 (emotional); Failure to perform procedure average payout: $386,851 (permanent), $131,559 (temporary), $56,251 (emotional); Unnecessary procedure average payout: $366,431 (permanent), $181,081 (temporary), $87,472 (emotional); Improper management average payout: $352,627 (permanent), $199,737 (temporary), $98,948 (emotional); Failure to obtain informed consent average payout: $305,295 (permanent), $117,938 (temporary), $88,445 (emotional); Wrong body part average payout: $262,656 (permanent), $84,921 (temporary), $37,210 (emotional); Retained foreign body average payout: $204,054 (permanent), $76,663 (temporary), $34,622 (emotional)	Improper performance; Improper technique; Failure to recognize complication; Retained foreign body; Improper management; Unnecessary procedure; Wrong body part surgery; Failure to obtain informed consent; Failure to perform procedure; Delay in performance
Gordhan CG et al. (33)	2015	USA	Colorectal surgery	Plaintiff verdict average award: $1,779,426.00 (range $180,000–$10,000,000); Settlement average award: $662,972.20 (range $67,500–$1,600,000); Failure-to-recognize complication subgroup plaintiff award mean: $1,347,564 (range $180,000–$5,219,086); Failure-to-recognize complication subgroup settlement mean: $851,333 (range $175,000–$1,600,000)	Failure to recognize complication in a timely manner; Damage to surrounding tissue during procedure; Postoperative infection; Anastomotic leak; Infection not recognized postoperatively; Injury to surrounding structures; Perforated colon; Misdiagnosis; Unnecessary procedure; Improper surgery; Lack of informed consent; Unsuccessful anastomosis; Fistula formation; Pulmonary embolism
Anandalwar SP et al. (34)	2017	USA	Pancreaticoduodenectomy (Whipple procedure); Pancreatic surgery	Plaintiff verdict average award: $3,366,652 (range $600,000–$10,300,000); Settlement average award: $398,333 (range $195,000–$500,000); Individual reported awards include: $10,300,000; $1,500,000; $1,066,608; $600,000; $500,000; $500,000; $195,000	Unnecessary pancreaticoduodenectomy; Misdiagnosis of pancreatic mass; Incorrect pathological diagnosis leading to surgery; Postoperative negligence; Failure to diagnose intra-abdominal abscess; Failure to diagnose postoperative hemorrhage; Failure to diagnose pulmonary embolism; Anastomotic leak; Portal vein injury; Mesenteric ischemia after vascular clamping; Retained surgical sponge; Inadequate preoperative work-up; Failure of informed consent; Postoperative bile leak; Sepsis following surgery; Hemorrhagic shock
Grayson CT et al. (35)	2018	USA	Hernia repair	Mean plaintiff award: $1,490,000 (median $575,000); Mean settlement award: $646,634 (median $498,171); Individual reported awards: $500,000; $350,000; $200,000; $2,000,000; $1,350,000; $650,000; $500,000; $8,178,200; $425,000; $150,000; $1,000,000; $442,000; $85,000; $498,171; $1,810,000	Improper performance; Failure of informed consent; Nerve injury; Chronic postoperative pain/neuralgia; Testicular injury; Vas deferens injury; Vascular injury; External iliac vessel injury; Bowel injury; Bladder injury; Infection; Retained foreign body; Missed malignancy; Retroperitoneal bleeding; Postoperative hemorrhage; Death following surgery
Tuchtan L et al. (36)	2016	France	Bariatric surgery	Civil judgment: €29,750 compensation to patient + €44,648 reimbursement to Primary Health Insurance Fund after gastric curvature perforation during LAGB; Civil case refusal of compensation for gastroesophageal fistula after VBG; Compensation pathway via French Conciliation and Compensation Commission (CCI) described; Expert transfer to insurer for compensation offer in 5 dossiers	Gastric perforation; Intestinal perforation; Anastomotic fistula; Gastroesophageal fistula; Bowel obstruction (dysphagia; slip; prolapse; stenosis; esophageal dilation); Vascular injury; Hemorrhage; Intra-abdominal infection; Abscess; Peritonitis; Pulmonary complications (pneumonia; pulmonary embolism); Postoperative pancreatitis; Gastric ischemia; Nutritional deficiency complications (Wernicke–Korsakoff syndrome); Surgical technical error; Delayed diagnosis of complication; Underestimation of postoperative symptoms; Delay in revision surgery; Failure of postoperative monitoring; Failure to follow national surgical recommendations; Organizational follow-up failure; Lack of informed consent; Conversion to different bariatric procedure without consent
Choudhry AJ et al. (37)	2016	USA	General surgery; Small bowel obstruction surgery; Emergency laparotomy; Adhesiolysis; Bowel resection for obstruction	Median overall award payout: $1,136,220 (range $29,575–$12,535,000); Median plaintiff verdict payout: $1,406,900; Median settlement payout: $1,043,100	Failure to timely diagnose small bowel obstruction; Delay in instituting treatment; Incorrect diagnosis (e.g., gastroenteritis); Failure to complete preoperative work-up; Medication error; Failure to transfer patient appropriately; Failure to monitor patient; Incomplete or incorrect surgical procedure; Unnecessary procedure; Damage to surrounding structures (bowel; ureter); Retained surgical sponge; Anaesthesia complication; Delay in treatment of postoperative complication; Postoperative infection; Postoperative bleeding/hypotension not addressed; Failure of hospital staff to follow physician orders; Communication failure; Nursing monitoring failure; Preventable pulmonary embolism/deep venous thrombosis; Inadequate postoperative care
Farooq A et al. (38)	2020	USA	Cholecystectomy	Median award payout: $500,842 (IQR $195,000–$848,852); Total paid claims cost: $22,226,074; Plaintiff verdict reached in 19.5% of cases; Settlements 12.6%	Procedural error; Failure to treat; Failure to diagnose; Failure to obtain informed consent; Failure to refer/order tests; Medication error; Misinterpretation of diagnostic tests; Unnecessary surgery; Wrongful death; Retained foreign body; Delay in treatment; Preoperative management delay; Intraoperative error; Postoperative care failure
Swonke ML et al. (39)	2020	USA	Thyroid surgery	Plaintiff verdict mean $2,156,128 (median $1,505,200; IQR $402,823–$3,797,000); Settlement mean $425,000 (median $425,000; IQR $225,000–$625,000); Overall indemnity mean $1,809,902 (median $900,000; IQR $309,645–$2,594,000)	Vocal cord paralysis; Recurrent laryngeal nerve injury (unilateral/bilateral); Parathyroid injury; Death; Cancer progression; Lack of informed consent; Permanent tracheostomy; Wound injury; Infection; Emotional distress; Brachial plexus injury
Magowan D et al. (40)	2023	UK	General surgery	Total damages £483,543,107; Total cost £851,558,930; Mean damages per claim £79,162; Mean total cost per claim £137,210; 6084 claims closed with damages	Failure/delay in treatment; Intraoperative problems; Failure/delay in diagnosis; Inadequate postoperative monitoring; Failure/delay in referral; Lack of preoperative evaluation; Surgical complication recognition failure
Gartland RM et al. (41)	2019	USA	Thyroid surgery	Plaintiff payout in 33% of claims; Median total incurred cost per paid claim $277,913 (IQR $87,343–$783,663); Total paid claim cost $26.5 million	Technical intraoperative error; Clinical judgment failure; Communication failure; Documentation failure; Behaviour-related factors; Recurrent laryngeal nerve injury (unilateral/bilateral); Postoperative hematoma; Incomplete surgery; Hypocalcemia; Wrong-side surgery; Skin/electrocautery injury; Failure to recognize complication
Wind J et al. (42)	2007	Netherlands	Laparoscopic surgery; Laparoscopic cholecystectomy; Diagnostic laparoscopy; Appendectomy; Hernia repair; Gastric banding; Gastric perforation surgery	NS	Retroperitoneal vascular injury; Intraperitoneal vascular injury; Epigastric vessel injury; Small bowel injury; Large bowel injury; Gastric injury; Veress needle injury; Trocar insertion injury; Adhesion-related injury; Device introduction error; Delayed diagnosis of injury; Failure of intraoperative recognition
Alkhaffaf B; Decadt B (43)	2010	UK	Hernia repair	Total litigation cost £7.35 million; Mean payout £51,099 (range £100–£371,706); Higher payouts: sexual dysfunction £85,467; chronic pain £81,288; visceral injury £64,465	Intraoperative error; Delay in recognition of complication; Substandard postoperative care; Consent-related issues; Misdiagnosis; Delay in surgical treatment; Anesthetic complication; Visceral injury; Vascular injury; Testicular/cord injury; Chronic postoperative pain; Retained foreign body; Recurrence; Death
Murphy BL et al. (44)	2018	USA	Breast surgery	Median payout overall $1,402,875 (IQR $557,450–$2,474,250); Plaintiff verdict median $1,485,000; Settlement median $862,500; General surgeon median payout $939,500 (IQR $577,800–$1,808,358)	Delay in diagnosis of breast cancer; Improperly performed procedure; Misdiagnosis; Physical intraoperative injury; Negligent postoperative care; Lack/insufficient informed consent; Retained foreign object; Wrong-side surgery; Failure to diagnose recurrence; Improper adjuvant therapy
Choudhry AJ et al. (45)	2013	USA	Appendectomy	Average plaintiff award $794,152 (range $1550–$15,400,000); Average settlement award $1,434,286 (range $40,000–$7,000,000)	Delay in diagnosis of appendicitis; Intraoperative negligence; Failure to completely remove appendix; Bowel perforation; Vascular injury; Trocar injury; Postoperative mismanagement; Failure to recognize bowel injury; Failure to follow pathology results; Inadequate informed consent; Sepsis; Peritonitis; Ruptured appendix
Choudhry AJ et al. (45)	2017	USA	Bariatric surgery	Median settlement $984,500 (IQR $317,662–$1,479,250); Median plaintiff verdict $2,000,000 (IQR $480,692–$4,855,000); Range $14,955–$183,719,510	Delay in diagnosis or management of postoperative complication; Anastomotic leak; Hemorrhage; Infection; Small bowel obstruction; Pulmonary embolism; Gastric/bowel perforation; Nutritional mismanagement; Respiratory compromise; Failure of monitoring; Premature discharge; Damage to surrounding structures; Improper procedure; Retained foreign body; Inappropriate patient selection; Inadequate preoperative evaluation; Consent failure
Kassir R et al. (46)	2020	France	Bariatric surgery	NS	Anastomotic fistula; Staple-line leak; Digestive perforation; Unrecognized gastrointestinal injury; Delay in diagnosis or treatment; Non-compliance with indications; Visceral injury outside operative target; Trocar injury; Small bowel injury; Aortic injury; Esophageal injury; Hospital-acquired infection; Retained foreign body; Pulmonary embolism; Vitamin deficiency due to inadequate follow-up; Failure of postoperative monitoring; Inadequate informed consent
Hartnett DA et al. (47)	2019	USA	Cholecystectomy	Mean plaintiff payment $723,844 ± $1,119,457; Mean settlement payment $1,350,000 ± $563,471; Death cases mean payout $2,669,413	Problematic intraoperative visualization; Incorrect anatomical identification; Bile duct injury; Hepatic duct injury; Intestinal perforation; Vascular injury; Failure to convert to open surgery; Improper intraoperative response; Improper postoperative treatment; Incorrect preoperative assessment; Failure to treat; Unnecessary surgery
Scurr JRH et al. (48)	2010	UK	Cholecystectomy	Settlements disclosed £5000–£470,000 and €50,000–€225,000; vascular injury £15,000–£75,000; pulmonary embolism death £35,000	Bile duct injury; Hepatic duct injury; Clip/transection injury; Diathermy injury; Hepatic artery injury; Postoperative bile leak; Delayed diagnosis of injury; Major vascular injury; Bowel injury; Duodenal injury; Postoperative hemorrhage; Failure to control bleeding; Failure to diagnose pulmonary embolism; Port-site hernia; Retained gallstones; Foreign body; Chronic pain
O'Connell RL et al. (49)	2021	UK	Breast surgery	Annual litigation cost £5.57 M–£9.59 M; Total cost £41.27 M; Highest individual claims £600,000–£1.2 M	Delay in diagnosis; Dissatisfaction with cosmetic outcome; Surgical decision-making error; Clinical judgment failure; Consent failure; Communication failure; Incomplete excision; Intraoperative injury; Retained foreign body; Implant complications; Surgical-site infection; Postoperative complication; Medication error; Delay in treatment
Weber CE et al. (50)	2013	USA	Bariatric surgery	Mean indemnity $94,526 (1990–1999); $377,151 (2000–2009); Morbid obesity mean payouts $114,177 and $366,695	Improper performance; Failure to recognize complication; Procedural error; Treatment error; Diagnostic error; Delay/timeliness error; Death-related claims
Ardito F et al. (51)	2023	Italy	Cholecystectomy	Mean compensation €90,500 (range €10,000–€600,000)	Delayed referral to tertiary center; Failed postoperative management; Attempts at repair before referral; Endoscopic mismanagement; Surgical mismanagement; Vascular injury; Severe bile duct injury; Delayed diagnosis
Anteby R et al. (52)	2021	USA	Pancreatic surgery	Median payout $783,304 (range $10,000–$3,431,183)	Failure to diagnose/treat postoperative complications; Wrongful death; Intraoperative negligence; Lack of informed consent; Incorrect diagnosis; Failure to obtain biopsy; Anastomotic leak; Abdominal infection; Hemorrhage; Pulmonary embolism; Retained foreign body
Walters AL et al. (53)	2013	USA	Hernia repair	Compensation range $19,000–$8,000,000	Retained foreign body; Failure of informed consent; Incorrect surgical technique; Unnecessary surgery; Infection; Bowel perforation; Esophageal injury; Nerve injury; Hemorrhage; Chronic pain

### a Types of surgery

Of the 41 studies in the table, the literature shows that medical liability claims in general surgery are disproportionately concentrated in specific “clusters” of procedures, with high-frequency procedures and procedures with a recognized risk of serious complications being predominant: bariatric surgery (6/41, 14.6%) [14], [18], [36], [45], [46], [50] and hernia repair (6/41, 14.6%) [17], [24], [27], [35], [43], [53] appear as the most frequent categories, followed by thyroid surgery (5/41, 12.2%) [15], [25], [31], [39], [41] and cholecystectomy (5/41, 12.2%) [28], [38], [47], [48], [51], while colorectal surgery (4/41, 9.7%) [16], [29], [30], [33] and breast surgery (2/41, 4.9%) [44], [49] are recorded less frequently but repeatedly. Furthermore, a portion of the studies is not limited to a single procedure but presents mixed samples of “general surgery” (e.g. multiple abdominal/emergency/laparotomy, laparoscopies, appendectomies, upper digestive tract procedures, hepatobiliary, etc.), as well as more specialized groups (e.g. retrograde/paraesophageal hernias, pancreatic surgery/Whipple, neuroendocrine tumors), which supports that these cases does not only concern “rare disasters”, but a wide range of everyday surgical activities, especially when technical complications or problems of timely recognition and treatment coexist [19], [20], [21], [22], [23], [26], [32], [34], [37], [40], [42], [52], [54].

### b Awarded/reported compensation payouts

Overall, quantitative compensation data, reported either as per-case awards, measures of central tendency (median or mean values), ranges, or total financial costs, were available in most of the included studies. The reported amounts showed substantial variability, largely driven by differences in legal frameworks, the severity of patient harm, and reporting methods, including jury verdicts, settlements, indemnity payouts, and aggregated institutional costs.

In the United States (USD), compensation figures included both central tendency estimates and extreme values. Reported median settlements reached $984,500 (IQR $317,662–$1,479,250), while median plaintiff verdicts were approximately $2,000,000 (IQR $480,692–$4,855,000). Across individual datasets, compensation ranged from $14,955 to $183,719,510 per case. Additional series reported median payouts of $783,304 (range $10,000–$3,431,183), with overall award ranges extending from $19,000 to $8,000,000. Some studies evaluated the broader economic burden rather than individual claims, describing inflation-adjusted cumulative payments of up to $5,866,435,277 over multi-year periods, with an average payout per claim of approximately $301,259. These figures reflect systemic financial impacts rather than typical compensation in individual cases [15], [28], [30], [32], [33], [34], [35], [37], [38], [39], [41], [44], [45], [47], [50], [52], [53], [54].

Similarly, European studies (EUR) demonstrated wide variability in the amount of awarded compensation. Maximum reported payments reached €1,724,000, while court awards as high as €1,421,636 were documented. Mean compensation values in individual series were reported at approximately €90,500 (range €10,000–€600,000). Case-specific awards included amounts such as €81,000 following biliary tract injury and €30,000 in fatal postoperative complications after appendectomy. Institutional estimates further indicated compensation levels of approximately €50,000 for permanent unilateral vocal cord paralysis and up to €200,000 for permanent bilateral injury, highlighting the influence of the severity of functional impairment on indemnity valuation [17], [18], [19], [23], [25], [36], [51].

In the United Kingdom (GBP), reported settlements ranged from £5000 to £470,000. Mean payouts per claim were approximately £51,099 (range £100–£371,706), with detailed analyses demonstrating variation according to causative factors, including delayed diagnosis (mean ∼ £61,106; up to £550,745), recurrent laryngeal nerve injury (mean ∼ £137,717; up to £945,685), and so-called “never events” (mean ∼ £22,673). As observed in the United States, several studies additionally reported large-scale cumulative expenditures, with total costs reaching £851,558,930 across thousands of claims, thereby illustrating the economic burden at a healthcare system level [24], [31], [40], [43], [48], [49].

Overall, although a proportion of malpractice claims do not result in financial compensation, successful claims, particularly those involving permanent disability, severe septic complications, or death, are associated with substantially high indemnity payments. The magnitude of compensation varies considerably across countries and surgical procedures, reflecting differences in medico-legal environments and compensation systems.

### c Causes of negligence/legal claims (causes)

The causes underlying legal claims demonstrate consistent and recurring patterns across the included studies, with litigation rarely arising from a single adverse event but rather from a sequence of clinical and organizational factors, typically involving an initial complication followed by delayed recognition and inadequate communication or documentation.

Technical errors and iatrogenic injuries represent the most frequently reported category (33/41 studies), including injuries to adjacent anatomical structures such as vascular, intestinal, or ureteral damage. Characteristic procedure-specific complications are repeatedly described, particularly bile duct injury following cholecystectomy and recurrent laryngeal nerve injury resulting in vocal cord paralysis after thyroidectomy. Additional contributing factors include intraoperative bleeding, inappropriate surgical technique, and performance of unnecessary or incorrect procedures [15], [16], [18], [19], [21], [22], [24], [25], [26], [27], [28], [29], [31], [32], [33], [34], [35], [36], [37], [38], [39], [40], [41], [43], [45], [46], [47], [48], [50], [51], [52], [53], [54].

Postoperative septic complications and failure-related events were also commonly reported (24/41 studies), encompassing perforations, intra-abdominal abscesses, peritonitis, and anastomotic or staple-line leaks. These complications appear prominently in bariatric and colorectal surgery and constitute a major source of malpractice claims, particularly when associated with delayed diagnosis or management [14], [16], [17], [18], [19], [22], [24], [26], [27], [28], [29], [31], [33], [34], [35], [36], [37], [45], [46], [47], [48], [52], [53], [54].

Delay in diagnosis or therapeutic intervention emerged as another central cause of litigation (25/41 studies), especially in emergency surgical settings or in cases with atypical clinical presentation [16], [18], [22], [24], [25], [26], [27], [29], [31], [32], [33], [34], [35], [38], [39], [40], [41], [43], [46], [47], [48], [50], [52], [53], [54]. Such delays were frequently linked to postponed imaging, delayed reoperation or referral, and underestimation of disease severity. Similarly, failure to promptly recognize postoperative complications and inadequate postoperative monitoring or management were reported in a considerable proportion of studies (13/41), often explicitly described as “failure to recognize complication” or “poor postoperative care” [14], [16], [17], [18], [19], [24], [26], [27], [35], [46], [47], [52], [53].

Non-technical factors were equally significant from a medico-legal perspective. Issues related to informed consent, physician–patient communication, and insufficient clinical documentation were identified in 19/41 studies and frequently acted as amplifying factors transforming a recognized surgical complication into a legal dispute [14], [15], [16], [18], [19], [20], [21], [25], [26], [27], [28], [30], [31], [32], [34], [37], [44], [45], [54]. Retained foreign bodies and similar events were repeatedly documented (19/41 studies), typically resulting in disproportionate legal consequences due to their preventable nature [16], [17], [18], [19], [21], [24], [26], [27], [30], [33], [34], [35], [36], [37], [44], [46], [47], [50], [53]. Finally, thromboembolic complications, including pulmonary embolism, were reported in 9/41 studies and were commonly associated with allegations of inadequate prophylaxis or delayed recognition, particularly in bariatric surgical populations [14], [18], [20], [32], [33], [34], [36], [37], [48].

## Discussion

This systematic review demonstrates that medical liability in general surgery is a long-standing and multifactorial problem with significant professional and economic implications for surgeons and healthcare systems worldwide. The findings of the analysis of the 41 included studies confirm that general surgery is among the specialties with increased legal exposure, which is directly related to the specialty's invasive nature, the need to make rapid decisions, and the management of patients with potentially life-threatening conditions.

The distribution of legal claims by type of procedure shows that most lawsuits involve routine procedures rather than rare or particularly complex ones, such as cholecystectomy, hernia repair, and appendectomy. This finding suggests that the frequency of a procedure is an important factor in legal exposure, as even low complication rates translate into an increased absolute number of cases. At the same time, higher-risk procedures, such as bariatric and colorectal surgery, show a disproportionate share of legal cases due to the severity of potential postoperative complications.

Analysis of the amounts awarded revealed particularly large variation across countries and legal systems, with the highest amounts recorded mainly in cases of permanent functional impairment, serious septic complications, or death. Although only a fraction of cases result in financial compensation, the amounts awarded in successful claims are often substantial, underscoring the significant financial impact of medical liability on surgical practice. The differences among compensation systems, insurance structures, and social perceptions of medical liability seem to largely explain the observed discrepancies [1], [55], [56].

Of particular interest is that most legal claims are not attributed solely to technical errors but to a combination of factors, including delayed diagnosis, failure to recognize complications in time, and inadequate postoperative follow-up. This sequence suggests that legal exposure in general surgery is often more related to the management of complications than to their initial occurrence. In addition, non-technical factors, such as inadequate patient information, incomplete consent documentation, and communication problems, emerged as determinants in the development of a complication into a legal claim.

These findings reinforce the view that preventing medical liability in general surgery is not limited to improving surgical technique alone but requires a multi-level approach that includes organizational interventions, clear follow-up protocols, early recognition of complications, and effective patient communication. Strengthening the safety culture, systematic training in complication management, and more complete documentation of medical practice may substantially reduce legal claims and curb defensive medicine [55], [57].

Adopting strategies to reduce defensive medicine can have both beneficial and potentially negative effects on patient health and care outcomes. On the positive side, reducing unnecessary tests, referrals, or procedures can reduce patient distress, limit overtreatment, reduce healthcare costs, and improve the quality of medical decisions. However, any attempt to reduce defensive practices should be implemented with caution, as an overly restrictive approach could increase the risk of delayed diagnosis and, consequently, delayed intervention. Therefore, the goal should not be to simply reduce diagnostic or therapeutic activity, but rather to promote evidence-based, well-documented, and patient-centered decision-making.

However, the results of this review should be interpreted with certain limitations in mind. The included studies show considerable heterogeneity in design, legal frameworks, and data reporting, which precluded quantitative synthesis or meta-analysis. In addition, the presence of multiple currencies and the lack of inflation adjustment limit the ability to compare economic data directly. Finally, much of the available literature comes from specific countries with developed legal record systems, which may limit the generalizability of the findings.

Despite these limitations, the consistency of findings across different geographic regions suggests that medical tort liability in general surgery is a global challenge with common recurring patterns. A systematic understanding of the causes of legal claims may help improve the quality of surgical care, reduce adverse events, and maintain trust between physicians and patients.

## Conclusion

This systematic review demonstrates that medical tort liability is a significant issue in general surgery, impacting both clinical practice and the financial burden on health systems. Most legal claims concern commonly performed procedures and arise mainly from delayed diagnosis, technical complications, and inadequate recognition or management of postoperative events, with factors such as informed consent and patient communication playing a crucial role.

Understanding the patterns that lead to legal claims can help improve patient safety, enhance the quality of surgical care, and reduce the need for defensive medicine practices.

## CRediT authorship contribution statement

**Ioannis Ketsekioulafis:** Writing – original draft, Investigation, Data curation. **Konstantinos Katsos:** Writing – review & editing, Formal analysis, Data curation. **Christoforos Kolentinis:** Writing – original draft, Investigation. **Theofano Sapka:** Writing – original draft, Investigation. **Chara Spiliopoulou:** Writing – review & editing, Formal analysis. **Theodoros Lytras:** Writing – review & editing, Formal analysis. **Emmanouil I. Sakelliadis:** Writing – review & editing, Supervision, Methodology, Formal analysis, Conceptualization. **Nikolaos Arkadopoulos:** Writing – review & editing, Supervision, Methodology, Formal analysis, Conceptualization.

## Clinical trial number

Not applicable.

## Consent for publication

Not applicable.

## Ethics approval and consent to participate

Not applicable.

## Funding sources

No funding was received.

## Declaration of competing interest

The authors have no competing interests to declare that are relevant to the content of this article.

## Data Availability

Not applicable.
